# Hepatoprotective Effects of Total Triterpenoids and Total Flavonoids from *Vitis vinifera* L against Immunological Liver Injury in Mice

**DOI:** 10.1155/2012/969386

**Published:** 2012-01-04

**Authors:** Tao Liu, Jun Zhao, Long Ma, Yusong Ding, Deqi Su

**Affiliations:** ^1^College of Public Health, Xinjiang Medical University, Urumqi 830011, China; ^2^Xinjiang Key Laboratory for Research and Development of Uighur Medicine, Institute of Materia Medica of Xinjiang, Urumqi 830004, China; ^3^Department of Public Health, College of Medicine, Shihezi University, Shihezi 832003, China

## Abstract

Suosuo grape (the fruits of *Vitis vinifera* L) has been used for prevention and treatment of liver diseases in Uighur folk medicine in China besides its edible value. In this study, the hepatoprotective effects of total triterpenoids (VTT) and total flavonoids (VTF) from Suosuo grape were evaluated in Bacille-Calmette-Guerin- (BCG-) plus-lipopolysaccharide- (LPS-) induced immunological liver injury (ILI) in mice. Various dose groups (50, 150, and 300 mg/kg) of VTT and VTF alleviated the degree of liver injury of ILI mice, effectively reduced the BCG/LPS-induced elevated liver index and spleen index, hepatic nitric oxide (NO), and malondialdehyde (MDA) content, increased liver homogenate alanine aminotransferase (ALT) and aspartate aminotransferase (AST) levels, and restored hepatic superoxide dismutase (SOD) activity in ILI mice. VTT and VTF also significantly inhibited intrahepatic expression of Th1 cytokines (IFN-**γ** and IL-2) in ILI mice and increased intrahepatic expression of Th2 cytokines (IL-4 and IL-10). Moreover, the increased Bax/Bcl-2 ratio was significantly downregulated by VTT and VTF in liver tissue of ILI mice. These results are comparable to those of biphenyl dicarboxylate (DDB, the reference hepatoprotective agent) and suggest that VTT and VTF play a protective role against immunological liver injury, which may have important implications for our understanding of the immunoregulatory mechanisms of this plant.

## 1. Introduction

Chronic hepatitis B (CHB) remains a major public health problem, despite the available effective vaccines. There are about 400 million people with hepatitis B virus (HBV) infection, who are at high risk of developing cirrhosis and/or primary hepatocellular carcinoma. Up to 30% of the chronic carriers will die of complications of these chronic liver diseases [1]. According to the HBV replication cycle and the host immune response, two major strategies can be applied to treat HBV infection: (i) regulation of the immune function with immunomodulatory agents such as alpha-interferon and pegylated interferon; (ii) inhibition of the viral replication at various stages including inhibition of HBV entry into the hepatocyte by interfering virus-cell interaction, inhibition of viral transcription and synthesis of viral proteins, or inhibition of HBV DNA polymerase activity [2–4].

 Grape (the fruit of *Vitis vinifera *L.) is a favorite food widely distributed to the world and also has been used to treat various diseases because of many biological activities in traditional medicine, such as diarrhea, hepatitis, and stomachaches [5]. Suosuo grape is a breed of Grape and mainly produced in Turpan, Shanshan, and Hetian regions of Xinjiang in China and has been used as a folk medicine for hepatitis and pedo-measles [6]. 

The active compounds of Grape include triterpenoids, flavonoids, and polysaccharides [7–9], and these compounds have been suggested to be responsible for the pharmacological activities observed with grape [10]. Previous studies showed that total triterpenoids (VTT), total flavonoids (VTF), and polysaccharides (VTP) from Suosuo grape could effectively suppress the secretion of HBsAg and HBeAg from HepG2.2.15 cells in a dose-dependent manner, as well as the HBV DNA, and the results of orthogonal design experiment showed that the combination of extracts (VTT 20 *μ*g/mL, VTF 50 *μ*g/mL and VTP 50 *μ*g/mL) had the best optimistic inhibitory effect on HBeAg secretion [11]. In addition, VTT had significant protective effects against immunological liver damage induced by Bacille Calmette-Guerin (BCG) plus lipopolysaccharide (LPS)* in vitro* [12].

Although anti-HBV and hepatoprotective activities of extracts from Suosuo grape have been verified by *in vitro* experiments, an *in vivo* study has not been published so far. Therefore, in this study, we aimed to further investigate the possible immunological mechanism of the hepatoprotective activity of VTT and VTF from Suosuo grape by BCG-plus-LPS-induced mice immunological liver damage.

## 2. Materials and Methods

### 2.1. Plant Material

Suosuo grape were collected from Tulufan, Xinjiang Uirghur autonomous region, in China, in May 2006. The plant materials were identified as Suosuo grape by Researcher Yan Fu Zhang, Institute of Materia Medica of Xinjiang. A voucher specimen was deposited at the Institute of Materia Medica of Xinjiang in China.

### 2.2. Chemicals

LPS (*E. coli* 0555:B5) was a product from Sigma (St. Louis, MO, USA). BCG vaccine was obtained from the Shanghai Institute of Biological Products. Biphenyl dicarboxylate (DDB, content more than 99% as determined by HPLC) was bought from Dezhou Deyao Pharmaceutical Co (Shandong, China). Assay kits for aspartate aminotransferase (AST) and alanine aminotransferase (ALT) were provided by Zhongsheng Tech (Beijing, China). Commercial kits used for determining lipid peroxidation, nitric oxide (NO), and superoxide dismutase (SOD) activity were obtained from the Jiancheng Institute of Biotechnology (Nanjing, China). Rat anti-mouse CD8-FITC and CD4-FITC monoclonal antibodies were from Caltag Co (Burlingame, CA, USA). Murine ELISA kits for IFN-*γ*, IL-2, IL-4, and IL-10 were from Biosource Co (Camarillo, CA, USA). Bax and Bcl-2 with mouse monoclonal antibodies were from Biosyn. Biotech. Co. (Beijing, China). All other chemicals were of analytical grade and commercially available.

### 2.3. Animals

The experiments were conducted on male Kunming strain mice (Experimental Animal Center of Xinjiang Medical University) weighing 20 ± 2 g. Animals were housed in plastic cages with a room temperature of 22 ± 1°C under a 12 h light-dark cycle and provided with rodent chow and water *ad libitum*. The studies were carried out in accordance with the current recommendations as described by the ethical guidelines for investigation and were approved by the Ethical Committee of Xinjiang Medical University (SYXK [Xin] 2003-0001).

### 2.4. Preparation of VTT and VTF

 Five kilograms of Suosuo grape was extracted with deionized water under reflux at 80°C for 6 h in several batches. The combined water extracts were evaporated under vacuum to specific gravity of 1.1-1.2, and ethanol about five times the original volume was added and kept at 4°C for 24 h to obtain precipitates. The gruffs were extracted with 95% ethanol under reflux at 80°C for 6 h in several batches. The extracts were combined, filtered, and evaporated under vacuum. The concentrated extracts were diluted with water and successively treated with petrol ether and EtOAc. Total triterpenoids (VTT) was prepared by the method of 95% ethanol recrystallized from the EtOAc fraction. The mother liquor of VTT crystalline powder combined with supernatant of ethanol precipitation to be separated by AB-8 resin. After deionized water eluting and cleaning impurities, 50% ethanol eluent was collected to afford total flavonoids (VTF). 

### 2.5. Quantification and Standardization of VTT and VTF

Chromatographic separation was achieved by using HPLC system consisting of a Shimadzu LC-10AD and YMC-Pack ODS-A column (150 × 4.6 mm, 5 *μ*m, with precolumn); the mobile phase consisted of methanol and H_2_O (86 : 14). Detection wavelength was 215 nm. The flow rate was 1.0 mL/min. Oleanolic acid content of VTT was 65.52 mg/100 mg. Total flavonoids content in the extracts was determined using described methods in Chinese pharmacopeia. Quantitation was based on the standard curve of rutin (0.2–1.0 mg/mL), dissolved in 95% ethanol. Flavonoids content was calculated with rutin as the standard and total flavonoids content of VTF was 35.28 mg/100 mg. 

### 2.6. Isolation and Identification of the Compounds

**Figure 1:**
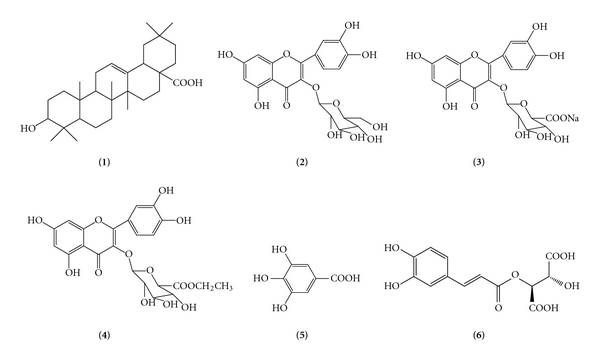
Chemical structure of compounds.

The VTT fraction was subjected to silica gel column chromatography and eluted with a gradient of Petrol ether/EtOAc, resulting in the isolation of oleanolic acid (**1**) [13], as a white powder, m.p. 291-292°C, from fraction eluted with Petrol ether/EtOAc (90 : 10, v/v). The chromatographic fractionation on AB-8 resin of the VTF fraction afforded a crude flavonoid from EtOH/H_2_O (50 : 50, v/v) eluate that was further purified by Sephadex LH-20 using MeOH as eluent repeatedly, yielding 130 mg of isoquercitrin (**2**) [14], as a yellow needle crystals, m.p. 232–234°C, 97 mg of quercitrin 3-*O-*β**-D-glucuronate sodium (**3**) [15], as yellow powder, m.p. 198.5–201.3°C, 13 mg of quercitrin 3-*O-*β**-D-glucuronopyranoside ethyl ester (**4**) [16], as yellow powder, m.p. 215–218°C, 7 mg of gallic acid (**5**) [17], as colorless needle crystals, m.p. 236–238°C, and 11 mg of caftaric acid (**6**) [18], as white powder, m.p. 123–125°C. The structures of the compounds were identified by their spectroscopic data (MS, ^1^H NMR, and ^13^C NMR), comparison with spectral data obtained from the literature, and co-TLC with authentic samples (Figure 1).

### 2.7. Experimental Procedure in Mice

Mice were randomly divided into nine groups (*n* = 12 per group). Immunological liver injury was induced via injection of a small dose of LPS into BCG-pretreated mice, as previously reported [19]. Briefly, 1 mg of BCG in 0.2 mL saline (approximately 5 × 10^7^ viable units per mouse) was injected via the lateral tail vein, with the exception of the control group, who received saline alone. VTT, VTF, and DDB were suspended in 0.5% sodium carboxymethylcellulose (CMC-Na) solution for administration. In the treatment groups, DDB (200 mg/kg·BW, as a reference drug), VTT (50, 150, or 300 mg/kg·BW), or VTF (50, 150 or 300 mg/kg·BW) was given daily via gavage for a period of 12 days. The normal group was given an equal volume of water. Twelve days later, all of the treated mice were given 10 *μ*g of LPS in 0.2 mL saline (control mice received saline alone) via lateral tail vein injection. Sixteen hours following LPS injection, blood samples were collected in glass tubes from orbital sinus for the analysis of CD4+/CD8+ and Th1 (IFN-*γ*, IL-2)/Th2 (IL-4, IL-10). Afterwards, all the animals were sacrificed by decapitation and livers and spleens were quickly excised freed from any adhering tissues and weighed. The liver and spleen weights of the mice/the wet weights (mg) per 10 g mouse × 100% were taken as the liver index (liver/body weight ratio), and spleen index (spleen/body weight ratio), respectively. Furthermore, some liver tissues were washed and perfused with chilled normal saline, minced and homogenized in an ice bath using a DY89-1-homogenizer (Ningbo Scientz Biotechnology Co, China, 3000 rpm for 10 min) in chilled normal saline to obtain 10% liver homogenate for the estimation of AST, ALT, NO, MDA, and SOD. Other liver tissues were placed in neutralized formalin before being stained and analyzed for pathological changes and the expression of bax/bcl-2.

### 2.8. Measurement of Liver Homogenate Contents of ALT, AST, SOD, MDA, and NO

Livers were homogenized, and the activities of AST and ALT and the MDA contents were determined using commercial kits. AST and ALT activities were determined as described previously [20] and expressed as an international unit per liter (U/L). MDA in liver tissue was determined by the thiobarbituric acid method [21]. Assays for total SOD activity were based on its ability to inhibit the oxidation of oxyamine by the xanthine-xanthine oxidase system [22]. The levels of NO in liver homogenate were assayed using the Griess reagent [23].

### 2.9. Quantification of Cytokine Concentrations in Plasma Samples

The concentrations of IFN-*γ* and IL-2, IL-4, and IL-10 in plasma samples were determined with specific ELISAs according to kit introduction.

### 2.10. Flow Cytometry

Liver lymphocytes were washed twice with Dulbecco's-PBS at pH 7.2 (Sigma) and once with RPMI 1640 medium (Sigma). Erythrocytes were lysed in 0.84% NH_4_Cl. Pure lymphocytes were resuspended at a final concentration of 1 × 10^6^ cells per 1 mL. After incubation at 4°C for 30 minutes, the cells were directly treated with rat anti-mouse CD4+-FITC- and CD8+-FITC-labeled monoclonal antibodies at 4 *μ*L per tube. Cell samples were washed three times in 0.1% sodium azide-PBS and analyzed by flow cytometry. Data for 10 000 cells, falling within FSC and SSC gates set for lymphocytes, were collected from each sample with a FACScan flow cytometer (Becton Dickinson Biosciences). All data files were analyzed with Cell Quest software.

### 2.11. Histological Examination

The liver tissue was collected and immediately fixed in 10% formalin, dehydrated in a graded ethanol (50–100%) series, cleared in xylene, and embedded in paraffin. Sections (4-5 *μ*m) were prepared and stained with hematoxylin and eosin (HE) dyes for photomicroscopic observations. For histological evaluation of the liver injury, areas of necrotic lesions were microscopically evaluated using an MCID Image analyzer. The degree of liver damage was categorized into five groups: Grade 0, no necrosis; Grade 1, spotty necroses around liver lobular central vein, interspersed inflammatory cell infiltration, and thermophilic acid body formation; Grade 2, necrotic area < 33.3%; Grade 3, necrotic area > 33.3% and < 66.7%; Grade 4, necrotic area > 66.7% [24]. The slides were scored independently by two pathologists who had no prior knowledge of their source. Each sample was observed at 100x magnification. The degree of liver damage was expressed as the mean of 8 different fields of view on each slide.

### 2.12. Assay for the Expression of Bcl-2 and Bax

Representative paraffin blocks were serially cut at 5 *μ*m thickness, deparaffinized in xylene, rehydrated in a graded ethanol series, and washed for 5 min with phosphate-buffered saline. For antigen retrieval, the sections were immersed in 10 mM citrate buffer (pH 6.0) and boiled. Endogenous peroxidase was blocked by incubation of the slides for 30 min with 3% hydrogen peroxide in methanol. Sections were then incubated for 24 h at 4°C with primary mouse monoclonal antibodies Bcl-2 (at 1 : 100 dilution) and Bax (at 1 : 100 dilution) at room temperature, respectively. The slides were tinctured with DAB and mounted according to SABC kits. A brown reaction indicated positive expression of Bcl-2 and Bax. The integral optical density (IOD) of Bcl-2 and Bax positive cells was determined.

### 2.13. Statistical Analysis

All values are presented as mean ± S.D. Statistical analysis of the data for multiple comparisons was performed by one-way analysis of variance (ANOVA) followed by Duncan's test (SPSS 12.0 software package). For a single comparison, the significance of differences between means was determined by the *t*-test. A level of *P* < 0.05 was accepted as statistically significant.

## 3. Results

### 3.1. Protective Effects of VTT and VTF on BCG-Plus-LPS-Induced ILI in Mice

**Figure 2:**
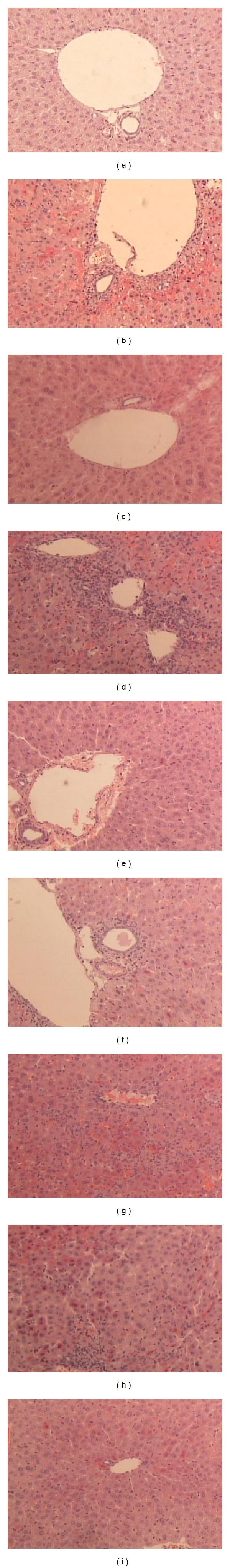
Histological image of liver tissues from ILI mice indicating the hepatoprotective effects of VTT and VTF. (a) Liver histopathologic examination showed no histological abnormalities in normal mice. (b) The liver tissue of ILI model mice, showing wide-range laminated necrosis, acidophilia changes, and portal infiltration. Significant hepatoprotective effects are seen in tissues primed with BCG and treated with (c) DDB (200 mg/kg), VTT at (d) 50, (e) 150, or (f) 300 mg/kg, or VTF at (g) 50, (H) 150, or (i) 300 mg/kg followed by LPS challenge. The degree of tissue damage was significantly reduced. The tissues were surgically excised and subjected to histological study by staining with hematoxylin and eosin. All the magnifications are ×100.

The liver index and spleen index were significantly increased in ILI mice.  Table 1 showed that the elevated liver index and spleen index were markedly reduced by daily VTT and VTF treatment with various doses (*P* < 0.01), and the reduction of liver homogenate ALT and AST levels was also significantly increased by daily VTT and VTF treatment with various doses (*P* < 0.01). Liver histopathologic examination showed no histological abnormalities in normal mice (Figure 2(a)). Liver cell wide-range laminated necrosis, acidophilia changes, and portal infiltration by inflammatory cells were seen after LPS injection in the BCG-primed mice (Figure 2(b)). The area, extent of necrosis, and degree of infiltration by inflammatory cells were substantially ameliorated by DDB (200 mg/kg), VTT (50, 150, or 300 mg/kg), and VTF (50, 150, or 300 mg/kg) treatments (*P* < 0.05) (Figures 2(c), 2(d), 2(e), 2(f), 2(g), 2(h), and 2(i) and Table 2). 

### 3.2. Effect of VTT and VTF on Liver Homogenate SOD Activity, MDA and NO Content in ILI Mice

Liver homogenate MDA content in ILI mice was significantly higher than that of the normal group, while total liver SOD activity was lower. After treatment with VTT and VTF at different doses (50, 150, or 300 mg/kg), liver homogenate SOD activity was restored significantly and MAD content was attenuated significantly. VTT and VTF (50, 150, or 300 mg/kg) also significantly inhibited the increased production of NO in liver homogenates of ILI mice (Table 3). The activities of middle-dose group of the two extracts (150 mg/kg) were comparable to those of DDB (200 mg/kg).

### 3.3. Influence of VTT and VTF on CD4+/CD8+ Cells in ILI Mice

**Figure 3:**
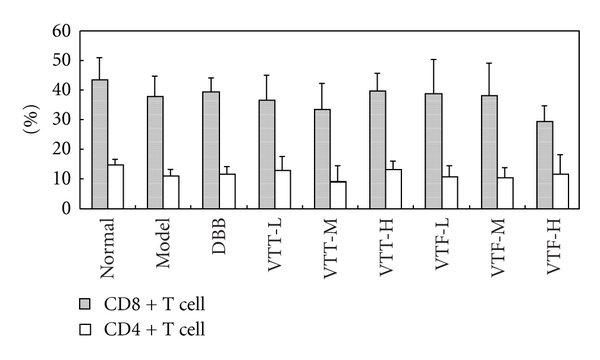
Effects of VTT and VTF on differentiation of T cells in ILI mice. The percentage of CD4+ and CD8+ cells in ILI cells was detected by flow cytometric assay using CD4 or CD8 monoclonal antibody conjugated with FITC (A). DDB, BCG + LPS + DDB (200 *μ*g/mL); VTT-L, BCG + LPS + VTT (50 mg/kg); VTT-M, BCG + LPS + VTT (150 mg/kg); VTT-H, BCG + LPS + VTT (300 mg/kg); VTF-L, BCG + LPS + VTF (50 mg/kg); VTF-M, BCG + LPS + VTF (150 mg/kg); VTF-H, BCG + LPS + VTF (300 mg/kg). *P* > 0.05 compared with normal group; *P* > 0.05 compared with model group.

Flow cytometric analysis showed no statistically significant differences in percentages of CD4+ and CD8+ cells, either among the groups or within the same group (*P* > 0.05) (Figure 3).

### 3.4. Influence of VTT and VTF on Th1/Th2 Cytokine Release in ILI Mice

The effects of VTT, VTF, and DDB on LPS-induced production of Th1/Th2 cytokines in plasma of ILI mice are shown in Table 4. In the ILI mice, the levels of Th1 cytokines IFN-*γ* and IL-2 were higher, while Th2 cytokines IL-4 and IL-10 levels were lower than in the normal group. After treatment with VTT and VTF (50, 150, or 300 mg/kg), middle- and high-dose group of both extracts can significantly inhibit production of IFN-*γ* and IL-2 and significantly increased levels of IL-4 and IL-10 (*P* < 0.01); especially the effect of high-dose group is comparable to that of DDB (200 mg/kg). In contrast, in the low-dose group, the effect on the ratio Th1/Th2 cytokines was weak.

### 3.5. Influence of VTT and VTF on the Expression of Bax/Bcl-2 Proteins in ILI Mice

**Figure 4:**
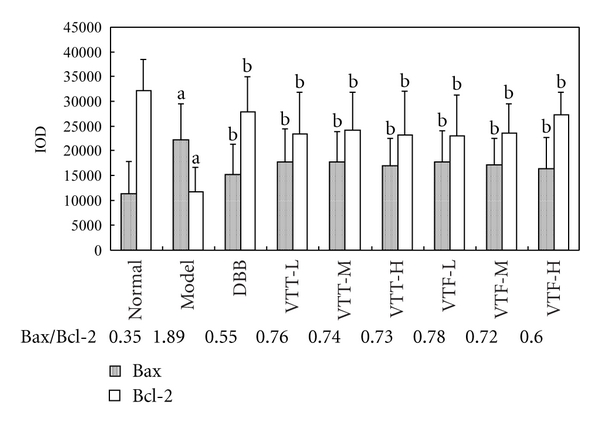
Influence of VTT and VTF on the expression of Bax/Bcl-2 proteins in ILI mice (*n* = 8). DDB, BCG + LPS + DDB (200 *μ*g/mL); VTT-L, BCG + LPS + VTT (50 mg/kg); VTT-M, BCG + LPS + VTT (150 mg/kg); VTT-H, BCG + LPS + VTT (300 mg/kg); VTF-L, BCG + LPS + VTF (50 mg/kg); VTF-M, BCG +LPS + VTF (150 mg/kg); VTF-H, BCG + LPS + VTF (300 mg/kg). ^a^ *P* < 0.01 compared with normal group; ^b^ *P* < 0.01 compared with ILI model group.

In the livers of ILI mice, the expression of Bax was increased in a dose-dependent manner compared to normal, and the expression of Bcl-2 was decreased in a dose-dependent manner compared to normal. The ratio of Bax/Bcl-2 in the livers of immunological hepatic injured mice was 1.89, which was higher than that of normal mice (0.35). The influence of VTF on Bcl-2 was more evident than that of Bax. Treatment with VTF, at all three doses, resulted in a significant increase in the expression of Bcl-2 and decreased the ratio of Bax/Bcl-2 to 0.78, 0.72, and 0.60, for VTF at 50, 150, or 300 mg/kg, respectively, of which the effect of high-dose group was near to DDB (200 mg/kg) (Figure 4). The effect of VTT was similar to VTF except that high-dose group was a little weak as follows: 0.76, 0.74, and 0.73, for VTT at 50, 150, or 300 mg/kg.

## 4. Discussion

The chemical analyses showed that VTT was rich in triterpenoids compounds and the content of oleanolic acid was 65.52%; VTF was rich in flavones compounds, and the content of total flavonoids was 35.28%. Both were mainly characteristic constituents from *Vitis vinifera *L, of which oleanolic acid is usually used as hepatoprotective drug. As a breed of *Vitis vinifera *L, Suosuo grape is used to treat various hepatitis in Uighur medicine, and its folk application was verified though the effect and mechanism of VTT and VTF on immunological liver injury mice in this study.

The model of immunological liver injury induced by BCG plus LPS is similar to the pathological course of chronic hepatic diseases in humans. In our study, the decrease of AST and ALT and the increase of MDA and NO levels in liver homogenates of ILI mice suggests enhanced lipid peroxidation leading to tissue damage and failure of antioxidant defense mechanisms. Treatment with VTT, VTF, and DDB (as a reference drug) during 12 days significantly prevented these changes. Moreover, VTT and VTF also significantly increased SOD contents of the liver homogenate. Hence, the mechanism of hepatoprotection of *Vitis vinifera *L may be due to its antioxidant effect. Histopathological studies showed that LPS/BCG caused fatty degeneration and necrosis of the liver tissue. Pretreatment with VTT and VTF exhibited protection, which confirmed the results of biochemical studies. These results indicate that treatment with VTT and VTF can protect the liver against LPS/BCG-induced hepatotoxicity.

Th1-type cytokines tend to produce the proinflammatory responses responsible for perpetuating autoimmune responses. Excessive proinflammatory responses can induce activation of CD8+ T lymphocytes (CTL), Kupffer cells, and hepatic macrophages. Activated CTL may aggravate hepatocyte apoptosis, while activated Kupffer cells and macrophages can provoke massive liver necrosis through microcirculatory disturbances, due to endothelial cell destruction and fibrin deposition in the hepatic sinusoids [25, 26]. The Th2-type cytokines include IL-4 and IL-10, which has more of an anti-inflammatory response. Th1 immunity is attenuated by the Th2 immune reaction [27]. The optimal scenario would therefore seem to be that humans should produce a well-balanced Th1 and Th2 response, suited to the immune challenge. Therefore, liver necrosis can develop as a consequence of any imbalance between Th1 and Th2 immune reactions in the liver [28]. In ILI mice, the significant immune balance disorders were showed as increase of Th1 cytokines (IFN-*γ* and IL-2) and decrease of Th2 cytokines (IL-4 and IL-10). 150 or 300 mg/kg dose group of VTT and VTF may have significantly inhibited intrahepatic expression of Th1 cytokines (IFN-*γ* and IL-2) and increased intrahepatic expression of Th2 cytokines (IL-4 and IL-10). Thus, both extracts from Suosuo grape could apparently produce a protective and ameliorating effect against liver tissue damage by regulating cytokines and restoring Th1/Th2 balance though could not promote proliferation and differentiation of immunological cells.

Apoptosis is an essential physiological process that plays a critical role in development and tissue homeostasis in multicellular organisms [29]. This study was to detect apoptotic cells in the liver tissues with the expression of Bcl-2 and Bax as proteins which are known to regulate this vital process. Bcl-2 itself functions as a repressor of apoptosis whereas another member of the family, bax, acts as a promoter of cell death. Abnormal apoptosis of hepatocytes is part of the pathological mechanism of acute or chronic hepatic injury. As such, investigation of the effects and mechanisms of liver-protecting drugs on apoptotic events is of obvious significance to liver injury research [30, 31]. The ratio of Bax/Bcl-2 may modulate or normalize the occurrence of apoptosis. Both a reduction in Bax expression and an increase in Bcl-2 expression were observed in Figure 4. VTT and VTF could be inhibiting the expression of Bax or promoting the expression of Bcl-2, or both, with the end result being a decrease in the Bax/Bcl-2 ratio in a dose-dependent manner.

In conclusion, the hepatoprotective effect of extracts from *Vitis vinifera *L (VTT and VTF) is related to its antioxidative, immunoregulatory properties and its ability to regulate hepatic apoptosis. The synergistic hepatoprotective effect of VTT, VTF, or other compounds from *Vitis vinifera *L will be subject to further study.

## Figures and Tables

**Table 1:** Effects of VTT and VTF on liver index, spleen index, and homogenate ALT and AST activities in ILI mice.

Group	*n*	Dose (mg/kg)	ALT (U/g prot)	AST (U/g prot)	Liver index (%)	Spleen index (%)
Normal	12		631.78 ± 81.08	215.73 ± 26.19	0.5564 ± 0.0577	0.0298 ± 0.0083
BCG + LPS	10		242.75 ± 63.15^a^	51.59 ± 12.03^a^	0.6979 ± 0.1238^a^	0.0843 ± 0.0247^a^
BCG + LPS + DDB	12	200	519.57 ± 154.05^b^	90.61 ± 13.30^b^	0.5466 ± 0.0628^b^	0.0502 ± 0.0101^b^
BCG + LPS + VTT	11	50	366.56 ± 83.77^b^	66.74 ± 18.56	0.5810 ± 0.0655^b^	0.0619 ± 0.0171^b^
	11	150	499.62 ± 96.11^b^	81.86 ± 14.86^b^	0.5801 ± 0.0725^b^	0.0646 ± 0.0116^b^
	12	300	507.19 ± 138.13^b^	96.69 ± 33.88^b^	0.5871 ± 0.0556^b^	0.0585 ± 0.0160^b^
BCG + LPS + VTF	10	50	408.70 ± 57.85^b^	63.63 ± 22.47	0.5524 ± 0.0908^b^	0.0581 ± 0.0213^b^
	12	150	489.24 ± 130.33^b^	79.97 ± 11.54^b^	0.5457 ± 0.0557^b^	0.0539 ± 0.0113^b^
	12	300	502.85 ± 129.12^b^	85.60 ± 18.40^b^	0.6090 ± 0.0643^b^	0.0552 ± 0.0092^b^

Values are the mean ± S.D. ^a^ *P* < 0.01, compared with normal group; ^b^ *P* < 0.01, compared with ILI model group.

**Table 2:** Effects of VTT and VTF on the pathological grading changes in ILI mice.

Group	Dose (mg/kg)	Degree of liver injury					*P* value
		0	I	II	III	IV	
Normal		8	0	0	0	0	
BCG + LPS		0	0	0	1	7	^#^
BCG + LPS + DDB	200	0	6	2	0	0	*
BCG + LPS + VTT	50	0	1	3	2	2	
	150	1	4	2	1	0	*
	300	1	3	3	1	0	*
BCG + LPS + VTF	50	1	1	1	3	2	*
	150	1	2	2	0	2	*
	300	1	2	4	1	0	*

Each group consists of 8 mice, and figures represent number of mice per grade. ^#^ *P* < 0.01, compared with normal group; *P < 0.01, compared with ILI model group.

**Table 3:** Effect of VTT and VTF on liver homogenate SOD activities, MDA and NO content in ILI mice.

Group	*n*	Dose (mg/kg)	SOD (U/mg prot)	MDA (nmol/mg prot)	NO (nmol/mg prot)
Normal	12		106.85 ± 14.25	14.71 ± 3.99	1.91 ± 0.30
BCG + LPS	10		57.67 ± 9.08^a^	32.49 ± 6.22^a^	4.27 ± 0.64^a^
BCG + LPS + DDB	12	200	92.84 ± 14.55^b^	20.45 ± 3.03^b^	2.26 ± 0.54^b^
BCG + LPS + VTT	11	50	76.02 ± 12.28^b^	25.52 ± 7.78^b^	3.41 ± 0.52^b^
	11	150	85.59 ± 12.07^b^	24.44 ± 2.39^b^	2.82 ± 0.56^bc^
	12	300	87.49 ± 11.98^b^	21.28 ± 4.86^b^	2.37 ± 0.40^bc^
BCG + LPS + VTF	10	50	62.91 ± 12.34	24.08 ± 4.87^b^	3.35 ± 0.68^b^
	12	150	93.14 ± 29.34^bc^	22.13 ± 2.26^b^	2.59 ± 0.44^bc^
	12	300	86.26 ± 10.83^bc^	20.76 ± 3.99^b^	2.34 ± 0.47^bc^

Values are the mean ± S.D. ^a^ *P* < 0.01, compared with normal group; ^b^ *P* < 0.01, compared with ILI model group. ^c^ *P* < 0.01, compared with low-dose group.

**Table 4:** Influence of VTT and VTF on Th1/Th2 cytokine release in ILI mice.

Group	*n*	Dose (mg/kg)	Th1		Th2	
			IL-2 (pg/mL)	IFN-*γ* (pg/mL)	IL-4 (pg/mL)	IL-10 (pg/mL)
Normal	12		29.77 ± 21.21	52.29 ± 23.31	19.91 ± 7.14	103.35 ± 23.43
BCG + LPS	10		61.10 ± 23.75^a^	116.13 ± 22.40^a^	11.75 ± 7.42^b^	47.11 ± 28.84^a^
BCG + LPS + DDB	12	200	32.48 ± 20.74^c^	68.00 ± 26.72^c^	17.17 ± 8.52	92.38±14.51^c^
BCG + LPS + VTT	11	50	63.59 ± 27.34	108.31 ± 33.16	12.20 ± 9.14	54.05 ± 20.43
	11	150	54.87 ± 20.85	94.01 ± 32.32	14.78 ± 8.87	67.77 ± 21.59^d^
	12	300	45.60 ± 26.79	72.50 ± 29.59^c^	15.55 ± 5.99	80.33 ± 21.71^c^
BCG + L PS + VTF	10	50	57.24 ± 28.74	104.44 ± 38.52	14.42 ± 6.64	57.73 ± 19.63
	12	150	43.95 ± 22.40	90.95 ± 29.24	15.47 ± 6.71	81.94 ± 19.04^c^
	12	300	36.78 ± 18.59^d^	78.43 ± 26.84^d^	15.78 ± 7.39	84.66 ± 23.64^c^

Values are the mean ± S.D. ^a^ *P* < 0.01, ^b^ *P* < 0.05, compared with normal group; ^c^ *P* < 0.01, ^d^ *P* < 0.01, compared with ILI model group.
